# Increasing Prion Propensity by Hydrophobic Insertion

**DOI:** 10.1371/journal.pone.0089286

**Published:** 2014-02-20

**Authors:** Aaron C. Gonzalez Nelson, Kacy R. Paul, Michelina Petri, Noe Flores, Ryan A. Rogge, Sean M. Cascarina, Eric D. Ross

**Affiliations:** Department of Biochemistry and Molecular Biology, Colorado State University, Fort Collins, Colorado, United States of America; Van Andel Institute, United States of America

## Abstract

Prion formation involves the conversion of proteins from a soluble form into an infectious amyloid form. Most yeast prion proteins contain glutamine/asparagine-rich regions that are responsible for prion aggregation. Prion formation by these domains is driven primarily by amino acid composition, not primary sequence, yet there is a surprising disconnect between the amino acids thought to have the highest aggregation propensity and those that are actually found in yeast prion domains. Specifically, a recent mutagenic screen suggested that both aromatic and non-aromatic hydrophobic residues strongly promote prion formation. However, while aromatic residues are common in yeast prion domains, non-aromatic hydrophobic residues are strongly under-represented. Here, we directly test the effects of hydrophobic and aromatic residues on prion formation. Remarkably, we found that insertion of as few as two hydrophobic residues resulted in a multiple orders-of-magnitude increase in prion formation, and significant acceleration of *in vitro* amyloid formation. Thus, insertion or deletion of hydrophobic residues provides a simple tool to control the prion activity of a protein. These data, combined with bioinformatics analysis, suggest a limit on the number of strongly prion-promoting residues tolerated in glutamine/asparagine-rich domains. This limit may explain the under-representation of non-aromatic hydrophobic residues in yeast prion domains. Prion activity requires not only that a protein be able to form prion fibers, but also that these fibers be cleaved to generate new independently-segregating aggregates to offset dilution by cell division. Recent studies suggest that aromatic residues, but not non-aromatic hydrophobic residues, support the fiber cleavage step. Therefore, we propose that while both aromatic and non-aromatic hydrophobic residues promote prion formation, aromatic residues are favored in yeast prion domains because they serve a dual function, promoting both prion formation and chaperone-dependent prion propagation.

## Introduction

Prions are protein-based infectious agents, caused by proteins capable of adopting an alternate, self-propagating amyloid-like structure. In mammals, misfolding of the prion protein PrP is responsible for the transmissible spongiform encephalopathies (TSEs), all of which are incurable and fatal [1]. Additionally, many other non-infectious diseases also involve the aggregation of proteins into amyloid deposits. In fungi, a number of proteins can adopt a prion state. The filamentous fungus *P. anserine* carries a prion protein, Het-S [2], that acts as part of a heterokaryon incompatibility mechanism. The yeast *Saccharomyces cerevisiae* carries at least nine proteins that convert to a prion state [3].

Yeast prions provide a useful model system for examining how amino acid sequence affects amyloid and prion propensity. For all but one of the amyloid-based yeast prion proteins, a glutamine/asparagine (Q/N) rich prion-forming domain (PFD) drives prion formation. Intriguingly, in the past few years, a number of proteins with prion-like domains (domains compositionally resembling the yeast PFDs) have been linked to various age-related degenerative disorders [4]: cytoplasmic inclusions containing FUS and TDP-43 are seen in both ALS and some forms of FTLD, and mutations in these proteins have been linked to some familial cases of ALS [5], [6], [7]; TAF15 and EWSR1 have separately been connected to ALS and FTLD [8], [9], [10]; mutations in hnRNPA1 and hnRNPA2/B1 cause IBMPFD/ALS (inclusion body myopathy with frontotemporal dementia, Paget’s disease of bone, and ALS; [11]); and mutations in TIA1 cause Welander distal myopathy [12]. A better understanding of how sequence and composition affect the amyloid propensity of prion-like domains would permit a better understanding of the mechanism of aggregation in these diseases. It would also allow for bioinformatics searches to identify new prion-like domains.

The yeast prion protein Sup35, which forms the [*PSI* ^+^] prion, is an essential subunit of the translation termination complex. Sup35 has three functionally distinct domains [13], [14], [15]. The N-terminal PFD (residues 1–114) is an intrinsically disordered domain that is necessary and sufficient for prion aggregation [13], [14], [15]. Like other yeast prions, it has high Q/N content and few hydrophobic residues [16]. The M domain (residues 114–253) is a highly charged, intrinsically disordered region that is not required for either prion formation or translation termination activity, but that stabilizes [*PSI* ^+^] [17]. The C domain (residues 253–685) is a structured region that is necessary and sufficient for translation termination.

Scrambling the PFD of Sup35 does not prevent prion formation, demonstrating that composition is a dominant variable affecting prion propensity [18]. A number of search algorithms to identify new prion proteins have been developed that take advantage of this fact by testing for compositional similarity to known PFDs [16], [19], [20]. Several prions were discovered using these methods [19], [21], [22]. However, this approach has limitations. Alberti *et al*. identified 100 yeast domains that had the greatest compositional similarity to existing PFDs and tested them for amyloid and prion-like activity using four different assays [19]. Remarkably, eighteen behaved as prions in all four assays. However, there was almost no correlation between the prion-forming ability of the 100 tested domains and their compositional similarity to existing PFDs [23], [24]. Therefore, while this algorithm is very effective at identifying prion candidates, it was ineffective at distinguishing among these candidates.

To better understand how composition affects prion propensity, we developed a method to quantify the prion-forming propensity of each amino acid in the context of a Q/N-rich PFD [24]. We replaced an eight amino acid segment from a scrambled Sup35 with a random library of sequences. We then selected for the subset of sequences that could form prions; the prion propensity of each amino acid was determined by comparing the frequency of occurrence of the amino acid among the prion-forming sequences to the frequency of the amino acid in the starting library. These prion propensity values were then used to build the prediction algorithm PAPA (Prion Aggregation Prediction Algorithm; [25], [26]).

PAPA is quite effective at discriminating between Q/N-rich domains with and without prion activity [24]. However, some of the individual prion propensity values for specific amino acids were quite surprising. As expected, charged residues and prolines were under-represented among prion-forming clones, consistent with their relative rarity in yeast PFDs [24]. Unexpectedly, Q/N residues were relatively neutral despite their prevalence in yeast PFDs, while hydrophobic residues, which are rare in yeast PFDs [16], were strongly over-represented among prion-forming clones, suggesting that they strongly promote prion activity.

This high predicted prion propensity for hydrophobic residues is particularly intriguing. The strong under-representation of hydrophobic residues in yeast PFDs would seem to suggest that these residues inhibit prion activity in the context of Q/N-rich domains. Indeed, any algorithm that uses compositional similarity to known PFDs to identify new prion proteins is predicated on the assumption that compositional changes that reduce the biases seen in known PFDs will reduce prion propensity; thus, such algorithms assume that increasing hydrophobic content will reduce prion propensity. At the same time, hydrophobic residues have long been thought to promote amyloid formation in the context of non-Q/N-rich proteins [27], although the applicability of these results to Q/N-rich proteins is unclear. Specifically, a number of algorithms, including Waltz [28], Zyggregator [29], ZipperDB [30], and TANGO [31], have been developed that can accurately predict the aggregation propensity of non-Q/N-rich domains; each of these algorithms favors hydrophobic residues, yet none of these algorithms are able to distinguish between Q/N-rich proteins with and without prion activity, making it unclear the extent to which results from non-Q/N-rich amyloid proteins can be applied to Q/N-rich proteins.

A recent study raised further doubts about the ability of hydrophobic residues to promote prion activity. Although expanded poly-glutamine tracts show high aggregation propensity, they do not propagate efficiently as prions in yeast because they are poorly fragmented by the chaperone machinery [32]; such fragmentation is required to maintain prions over multiple generations of cell division. Alexandrov *et al*. recently showed that insertion of aromatic residues into poly-Q tracts promotes fiber fragmentation, but that non-aromatic hydrophobic residues do not exert the same positive effect [33].

There are a number of possible hypotheses that could explain why non-aromatic hydrophobic residues are so rare in yeast PFDs and fail to promote prion activity when inserted into poly-Q tracts, yet showed high prion propensities in the screen used to develop PAPA. The simplest explanation is that the predicted prion-propensity values are either an artifact of the region tested or simply inaccurate. For example, because the prion propensity values for each amino acid were derived by random sampling, these values have large confidence intervals, so the non-aromatic hydrophobic residues may simply be less prion-prone than we predicted [24]. However, we hypothesized a more nuanced explanation. Aggregation and prion maintenance are distinct activities that appear to have distinct compositional requirements [34]. The Alexandrov experiments focused on prion maintenance. By contrast, the PAPA scores do not separate these two activities, so likely reflect some combination of the two. While non-aromatic hydrophobic residues appear unable to promote fiber fragmentation, they may still promote prion formation; in this case, aromatic hydrophobic residues may simply be favored in yeast PFDs because they can serve a dual role, promoting both prion formation and prion maintenance. To test this hypothesis, we specifically examined the effects of non-aromatic hydrophobic residues on prion formation by Sup35. We found that non-aromatic hydrophobic residues can promote prion formation to a remarkable degree. These results, combined with bioinformatics analysis of prion and non-prion Q/N-rich domains, provide insight into a number of unanswered questions about the sequence basis for prion activity.

## Materials and Methods

### Strains and Media

Standard yeast media and methods were used, as described previously [35], except that yeast extract-peptone-dextrose (YPD) contained 0.5% yeast extract instead of the standard 1%. In all experiments, yeast were grown at 30°C. Experiments were performed with *Saccharomyces cerevisiae* strain YER632/pJ533 (α *kar1*-*1 SUQ5 ade2*-*1 his3 leu2 trp1 ura3 sup35*::KanMx [*psi* ^−^] [*PIN* ^+^]; pJ533 expresses *SUP35* from a *URA3* plasmid as the sole copy of *SUP35* in the cell), a [*psi* ^−^] version of 780-1D/pJ533 [36].

### Design of the Mutants

For the hydrophobic insertions, the Excel random number function was used to select positions for insertion between amino acids 8–24 of Sup35. In each case, an equal number of isoleucines and valines were inserted. For the tyrosine deletions, the Excel random number function was likewise used to select which tyrosines should be deleted.

### Cloning

CEN plasmids expressing full-length Sup35 mutants from the *SUP35* promoter were generated using homologous recombination. The mutations were inserted into the N domain of *SUP35* in two steps. For each mutant, two PCR reactions were set up. The N-terminal portion of *SUP35* was amplified with EDR302 and a mutant-specific primer, while the C-terminal portion of *SUP35* was amplified with EDR262 and a second mutant-specific primer (see Table S1 for a complete list of primer sequences). Products of these two reactions were combined and reamplified with EDR301 and EDR262. The final PCR products were co-transformed with HindIII/BamHI-cut pJ526 [37] into yeast strain YER632/pJ533. Transformations were selected on SC-Leu, and then transferred to FOA plates to select for loss of pJ533.

To generate induction plasmids, the NM domain of each mutant was amplified by PCR using primers EDR1008 and EDR1084. EDR1084 installs a stop codon and XhoI restriction site at the end of the middle (M) domain, while EDR1008 installs a BamHI restriction site before the Sup35 start codon. PCR products were digested with BamHI and XhoI, and then inserted into BamHI/XhoI cut pKT24, a *TRP1* 2µm plasmid containing the *GAL1* promoter [37]. Ligation products were transformed into *Escherichia coli* and analyzed by DNA sequencing.

To generate vectors expressing GFP fusions, first the cassette containing the *GAL1* promoter and *ADH1* terminator was amplified from pKT24 using primers EDR1747 and EDR1748, which install SphI and EcoRI sites, respectively. This product was digested with SphI and EcoRI and then inserted into SphI/EcoRI cut YEplac112 [38] to generate plasmid pER687. Yeast-optimized GFP was then amplified from pYGFP [39] using primers EDR1898 and EDR1899, which add BamHI and SalI restriction sites, respectively, to the 5′ and 3′ ends of GFP. PCR products were digested with BamHI and SalI, and then inserted into BamHI/XhoI cut pER687, generating plasmid pER760. The NM domain of the Sup35 mutants were then amplified with EDR1008 and EDR1924, which add BamHI and XhoI restriction sites, respectively, to the 5′ and 3′ ends of the Sup35 NM. PCR products were digested with BamHI and XhoI, and then inserted into BamHI/XhoI cut pER760.

### Western Blot

Western blots were performed as previously described ([40]), using a monoclonal antibody against Sup35’s C-terminal domain (BE4 [41], from Cocalico Biologicals, kindly made available by Susan Liebman).

### [*PSI^+^*] Formation

For all prion formation assays except those for the hydrophobic rearrangement constructs, strains were transformed with either pKT24 or with a derivative of pKT24 in which the respective PFD was inserted under control of the *GAL1* promoter. Strains were grown for 3 days in galactose/raffinose dropout medium lacking tryptophan to select for pKT24 or the pKT24 derivative. It is not necessary to maintain selection for the plasmid expressing the full-length Sup35 mutant, because this plasmid expresses the only copy of *SUP35* in the cells, and *SUP35* is an essential gene. Serial 10-fold dilutions were spotted onto SC-ade medium to select for [*PSI* ^+^] cells and grown for 5 days. Although new colonies will continue to appear after 5 days, we find that these colonies tend to be unable to propagate the Ade^+^ phenotype when removed from selection.

The hydrophobic rearrangement constructs were only tested under uninduced conditions. These strains were grown in YPAD for 2 days, and then serial 10-fold dilutions were spotted onto SC-ade medium to select for [*PSI* ^+^] cells and grown for 5 days.

### Protein Expression and Purification

The NM domain of Sup35 was recombinantly expressed on a pET-17b expression vector in BL21 CodonPlus competent cells (Agilent Technologies, CAT#230245). A one liter 2×YT culture was grown to an OD600 of 1.0. Cells were induced with 0.5 mM IPTG for four hours. Cultures were centrifuged and pellets stored at −70°C. Protein was purified under denaturing conditions in two steps. First, cells were resuspended in lysis buffer (6 M GuHCl, 0.1 M KH_2_PO_4_, 10 mM Tris Base, 0.05% Tween 20, pH 8) and lysed by sonication. The lysate was loaded onto a Ni-NTA sepharose column (GE Healthcare, 17-5286-01). Sup35NM was eluted with imidizole buffer (6 M Urea, 0.1 M KH_2_PO_4_, 10 mM Tris Base, 0.05% Tween 20, 0.5 M Imidazole, pH 8). Second, fractions containing protein were pooled and diluted 1∶4 into loading buffer (6 M Urea, 50 mM MES pH 6.0). Sup35NM was loaded onto an SP Sepharose Ion exchange column and eluted in high salt (6 M Urea, 50 mM MES pH 6.0, 1 M NaCl). Fractions containing Sup35NM protein were pooled and concentrated using an Amicon Ultra centrifugal filter (Fisher, UFC901008). Protein was stored in urea at −70°C.

### *In vitro* Amyloid Aggregation Assay

The *in vitro* assays were performed using a protocol adapted from Collins *et al*. [42]. Briefly, reactions were set up as follows: A 96-well plate (Fisher, 07-200-567) was treated with 5% casein solution for five minutes at room temperature, then rinsed with DI water and allowed to dry. Protein and thioflavin-T stock solution were diluted to a final concentration of 5 and 25 µM, respectively, in 50 mM glycine buffer, with a final reaction volume of 200 µl. Fluorescence was monitored in a Victor3 Perkin Elmer fluorescence plate reader, with excitation and emissions wavelengths of 460 and 490 nm, respectively. Reactions were monitored for 48 h. Between readings, reactions were incubated without agitation for 3 minutes, and then shaken for 10 sec. The fraction aggregated was calculated by normalizing relative to the final fluorescence of the well.

### Bioinformatics Analysis of the Yeast Proteome

The complete set of systematically-named *Saccharomyces cerevisiae* open reading frames was downloaded from the Saccharomyces Genome Database (http://downloads.yeastgenome.org/sequence/S288C_reference/orf_protein/). To generate a histogram of the compositional distribution of the yeast proteome, the proteome was scanned using a 100 amino acid window size, scoring the amino acid composition of each window.

## Results

### Insertion of Hydrophobic Residues Increases Prion Formation

**Figure 1:**
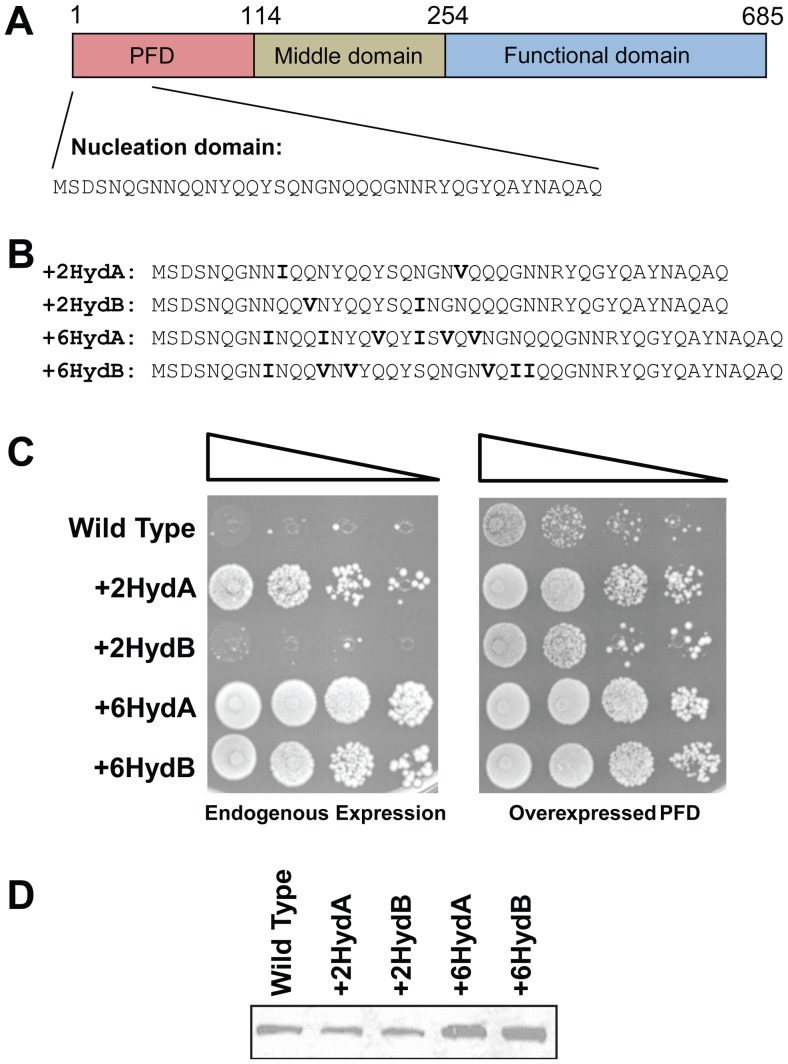
Insertion of hydrophobic residues increases prion formation. (A) Schematic of Sup35. The sequence of the nucleation domain (amino acids 1–40) is shown. (B) Sequences of the nucleation domains of each of the hydrophobic-addition constructs. Inserted hydrophobic residues are indicated in bold. For each, the remainder of the protein is the same as wild-type Sup35. (C) Prion formation by each construct. Strains expressing the indicated Sup35 mutants as the sole copy of Sup35 were transformed either with an empty vector (left) or with a plasmid expressing the matching Sup35 mutant under control of the *GAL1* promoter (right). All strains were cultured for three days in galactose/raffinose dropout medium, and then 10-fold serial dilutions were plated onto medium lacking adenine to select for [*PSI* ^+^]. (D) Western blot of wild-type and mutant Sup35.

Various studies suggest that a key difference between aromatic and hydrophobic residues in the context of Q/N-rich domains is that aromatic residues facilitate the chaperone-dependent fragmentation that is required for prion maintenance [33], [43]. However, the relative effects of aromatic and hydrophobic residues on prion formation are less clear. To specifically focus on the effects of hydrophobic residues on prion formation, we took advantage of the fact that the prion formation and maintenance activities of the Sup35 PFD largely reside in separate regions of the PFD [44]. The first 40 amino acids are highly enriched in Q/N residues and are required for prion nucleation and fiber growth, while amino acids 40–114 are thought to be primarily involved in prion maintenance [34], [44], [45], [46], [47]. To test the effect of non-aromatic hydrophobic residues on prion formation, we generated four constructs in which we inserted isoleucine or valine at random positions between residues 8–24 of Sup35, a region of the nucleation domain that is particularly important for prion activity [45]. Isoleucine and valine were chosen because they score as the most prion-promoting non-aromatic amino acids according to PAPA; leucine actually scores as slightly prion-inhibiting, likely due to its low β-sheet propensity [24]. Four *SUP35* mutants were generated (Figure 1A, B): two in which two hydrophobic residues were inserted into random locations in the nucleating domain of Sup35p (called +2HydA and +2HydB), and two in which six hydrophobic residues were inserted (+6HydA and +6HydB).

Each mutant was cloned into a CEN plasmid under the control of the *SUP35* promoter. These plasmids were shuffled into a yeast strain that lacks an endogenous copy of *SUP35*, but carries a maintainer copy expressed from a *URA3* plasmid. After selection for loss of the maintainer plasmid, strains were tested for their propensity to convert to [*PSI* ^+^]. [*PSI* ^+^] was detected by monitoring nonsense suppression of the *ade2-1* allele [48]. [*psi−*] *ade2-1* mutants cannot grow in the absence of adenine and form red colonies in the presence of limiting adenine due to accumulation of a pigment derived from the substrate of Ade2. However, [*PSI* ^+^] allows for occasional read through of the *ade2-1* nonsense mutation. Thus, [*PSI* ^+^] cells can grow in the absence of adenine, and grow white in the presence of limiting adenine.

Insertion of hydrophobic residues substantially increased the frequency of Ade^+^ colony formation (Figure 1C). Spontaneous wild-type prion formation is an extremely rare event, occurring in approximately one cell per million when Sup35 is expressed at endogenous levels [49]. Efficient [*PSI* ^+^] formation requires PFD overexpression, which increases the pool of soluble protein, thereby increasing the probability of the nucleation events that initiate prion formation (Figure 1C, right versus left panel) [50]. By contrast, the addition of six hydrophobic residues generated strains that appeared to be constantly [*PSI* ^+^], even in the absence of PFD overexpression (Figure 1C, left panel). This prion-promoting effect was so strong that the cells were even able to form [*PSI* ^+^] in the absence of [*PIN* ^+^], a prion required for wild-type Sup35 to form prions (Figure S1A) [51]. Even just two additional hydrophobic residues caused a significant increase in frequency of Ade^+^ colony formation, with roughly one in ten cells expressing the +2HydA construct forming Ade^+^ colonies in the absence of PFD overexpression (Figure 1C, right versus left panel). This increase was not due to changes in protein levels; although the +6Hyd constructs both showed modestly higher protein levels by western blot than wild-type Sup35, protein levels for the +2Hyd constructs were similar to wild-type Sup35 (Figure 1D).

Despite having identical amino acid compositions, the +2HydA and +2HydB constructs showed substantial differences in frequency of prion formation. This supports the idea that while amino acid composition is the dominant factor affecting prion propensity, primary sequence also exerts an effect [18], [19], [37].

To confirm that the Ade^+^ colonies were due to prion formation, Ade^+^ isolates from each mutant were tested for stability and curability. Guanidine hydrochloride cures yeast prions by disrupting the activity of Hsp104 [52], [53], a chaperone protein involved in prion propagation [54], [55], [56]. Individual Ade^+^ isolates were streaked on YPD, with and without the addition of 4 mM guanidine. Cells were then tested for loss of [*PSI* ^+^] by re-streaking onto medium containing limiting adenine (Figure S2). The majority of the Ade^+^ isolates from each of the hydrophobic addition constructs were stably Ade^+^ in the absence of guanidine, but lost the Ade^+^ phenotype after growth on guanidine (Figure S2), demonstrating that the phenotype was the result of a prion. Therefore, addition of hydrophobic residues dramatically increases the frequency of prion formation, without interfering with prion propagation.

Interestingly, some of the constructs rapidly reverted to the [*PSI* ^+^] after curing. The most extreme was the +6A construct. It formed predominantly weak prions, as indicated by a pink phenotype. Although these cells were fully red on guanidine medium (data not shown), upon restreaking onto non-selective medium, they rapidly converted to a mixture of red, white, pink, and sectored colonies.

**Figure 2:**
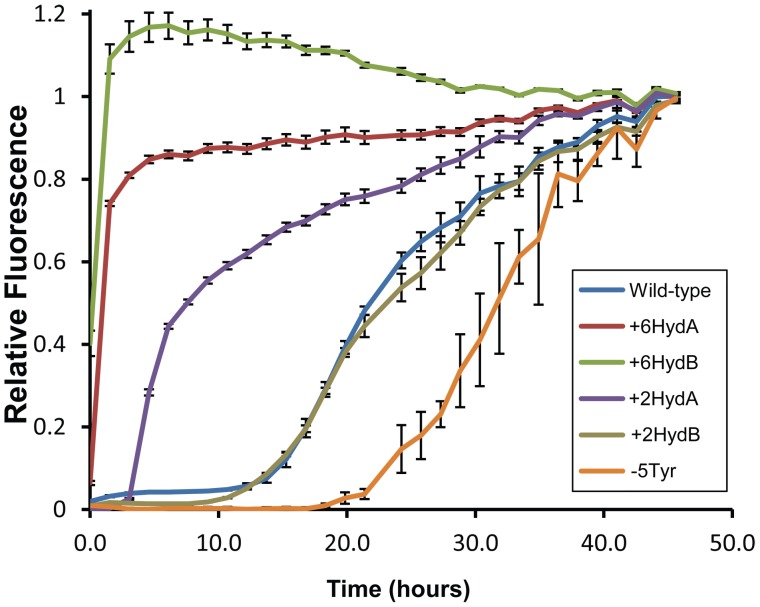
*In vitro* amyloid aggregation of the mutant prion forming domains. Aggregation of purified PFDs was monitored using thioflavin T. Reactions were incubated with intermittent shaking for 48 h. Fluorescent readings were taken approximately every 90 min. Error bars represent the standard deviations of three samples.

To ensure that the observed differences in prion formation were due to changes in prion propensity, rather than an artifact such as mislocalization, differences in toxicity, or alteration of a prion-modifying protein-protein interaction, the mutants were purified and assayed for amyloid formation *in vitro* (Figure 2). Amyloid aggregation was monitored using Thioflavin T, a dye that forms fluorescent complexes with amyloid fibrils, but not with soluble proteins or amorphous aggregates [57]. In each case, the rate of aggregation *in vitro* correlated well with prion formation *in vivo* (Figure 1C). As expected, wild-type Sup35 had a lag phase lasting approximately 9 hours before aggregation and increased in a roughly sigmoid fashion. Remarkably, +6HydA and +6HydB each showed no detectable lag phase and plateaued within three hours.

### The Effect of Primary Sequence on Prion Formation

Both +2HydA and +2HydB carry an extra isoleucine and valine. The observed prion formation differences between these compositionally identical constructs demonstrate that small changes in primary sequence can exert substantial effects on prion formation. Therefore, these constructs provide a useful system to explore the basis for such primary sequence effects.

**Figure 3:**
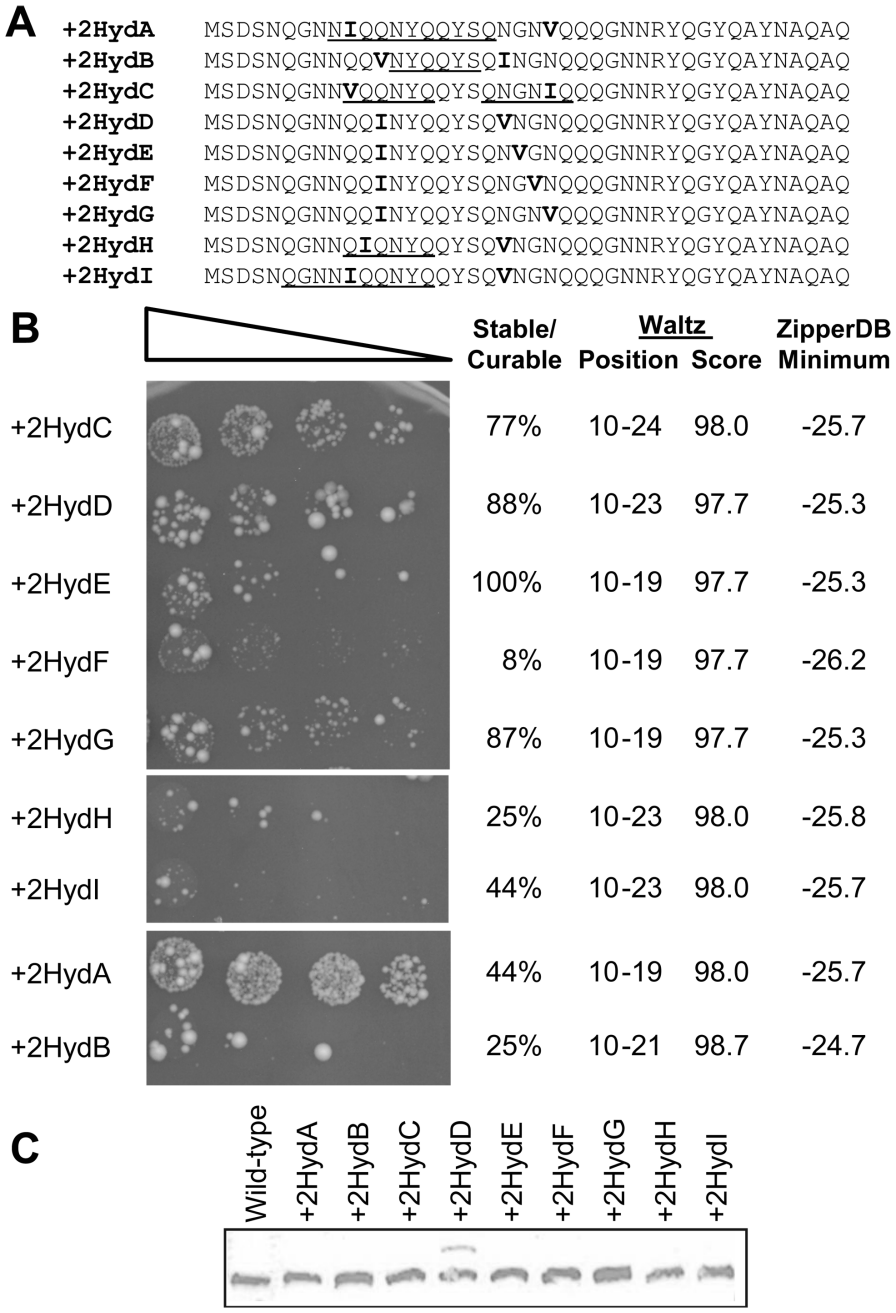
Effects of primary sequence on prion formation. (A) Amino acid sequences of constructs in which two additional hydrophobic residues were added at various positions within the Sup35 nucleation domain. For each, the remainder of the protein is the same as wild-type Sup35. Amyloid stretches, as predicted by Lopez de la Paz and Serrano [62], are underlined. The inserted hydrophobic residues are indicated in bold. (B) Prion formation by each of the constructs. Strains expressing the indicated Sup35 mutants as the sole copy of Sup35 were grown in YPAD medium for two days, and then 10-fold serial dilutions were plated onto medium lacking adenine to select for [*PSI* ^+^]. For each construct, the position and scores of amyloid stretches predicted by Waltz [28], as well as the minimum ZipperDB score [63], are indicated. Individual Ade^+^ colonies we picked from each plate and tested for stability and curability, as in Figure S2. Colonies were considered stable and curable if they maintained a white/pink phenotype on YPD, but were red after treatment with guanidine HCl. (C) Western blot of expression levels of wild-type and mutant Sup35s.

However, systematically repositioning the isoleucine and valine did not reveal any clear trend (Figure 3A,B). The constructs showed substantial differences in both the number of Ade^+^ colonies observed and the fraction of these colonies that propagated as stable, guanidine-curable prions (Figure 3B). Western blot showed only small expression differences among the mutants, and neither the frequency of Ade^+^ colony formation nor the stability of the Ade^+^ phenotype consistently correlated with expression levels (Figure 3C). Unexpectedly, the +2HydD mutant showed two bands: a predominant band at the expected size, and a minor band running at a higher molecular weight. This raises the possibility that a subset of the +2HydD protein pool could be undergoing modification, suggesting that prion formation levels for this mutant should be interpreted with caution.

These large differences in prion activity are not predicted by any of the commonly-used aggregation prediction algorithms. Not surprisingly, composition-based algorithms such as PAPA or Zyggregator were not effective at distinguishing among these constructs. However, while the prion propensity of Q/N-rich domains is predominantly determined by amino acid composition, a variety of evidence suggests that short sequence motifs may play a critical role in nucleating prion formation by Sup35 [58], [59], [60]. Thus, the mutations may affect prion activity by creating or disrupting amyloid-promoting primary sequence motifs. The Serrano group has used both computational and experimental techniques to determine a consensus hexameric sequence that promotes amyloid formation [61], [62]. Interestingly, the only such stretch in the nucleation domain of Sup35 overlaps with the region mutated in these constructs (Figure 3A). However, there did not appear to be any correlation between the presence of such stretches and prion activity (Figure 3A,B).

Other prediction algorithms were no more effective. A recent, more comprehensive study has expanded the definition of the hexameric amyloid stretch [28]. The prediction algorithm Waltz utilizes this broader definition and provides quantitative scores for different stretches. However, no correlation was seen between Waltz scores and Ade^+^ colony formation. All of the proteins had Waltz-positive segments; although there were differences in the length of these segments, there was no clear correlation between the length or score of the predicted amyloid stretch and observed prion activity. The same was true using the “High Specificity” setting, which is intended to reduce false positives; again, all constructs had Waltz-positive segments overlapping with the mutated region, and there was no correlation between the length of the predicted amyloid stretch and observed prion activity (data not shown).

Similar results were seen for ZipperDB, another algorithm that looks for 6-amino-acid aggregation-prone segments. ZipperDB is a structure-based prediction method. Sequences are threaded into a known NNQQNY amyloid-forming hexapeptide crystal structure and the energetic fit is determined [30], [63]. Segments with a free energy below −23 kcal/mol are considered to have high fibrillation propensity; insertion of a single such sequence into a loop region of RNase A was sufficient to cause amyloid formation [64]. All of the +2Hyd constructs had segments well below −23 kcal/mol that overlapped with the mutated region; however, there was no correlation between the predicted free energy and the observed frequency of prion activity (Figure 3B). In short, while it is clear that primary sequence effects do exist, none of the commonly used amyloid prediction algorithms successfully predict these effects.

### Deletion of Tyrosine Residues Reduces Prion Formation and Aggregation

**Figure 4:**
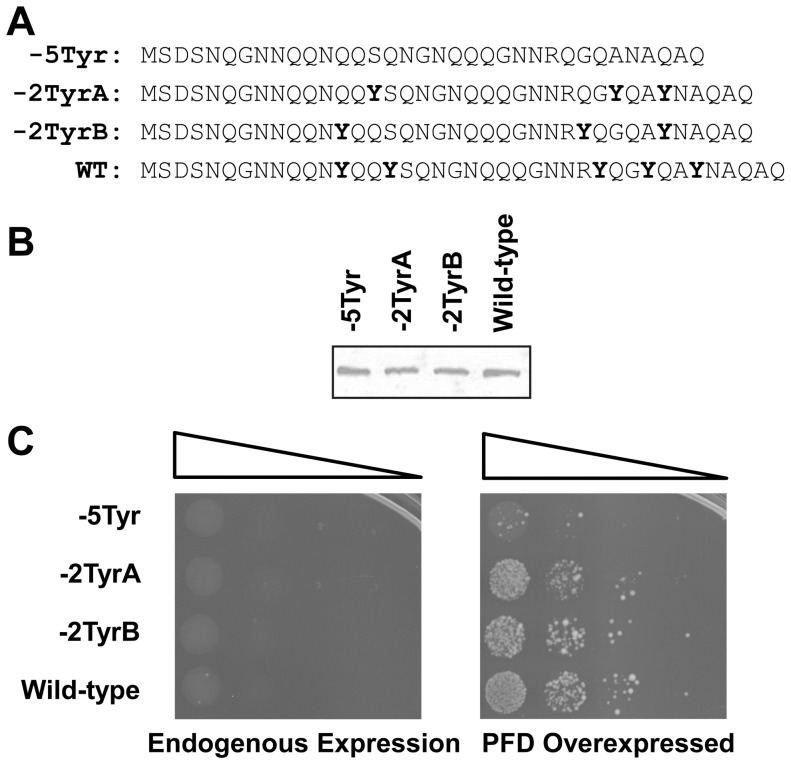
Deletion of tyrosine residues reduces prion formation. (A) Amino acid sequences of constructs in which tyrosines were deleted from various positions within the Sup35 nucleation domain. Tyrosines are indicated in bold. (B) Western blot of expression levels of wild-type and mutant Sup35s. (C) Prion formation by each construct. Strains expressing the indicated Sup35 mutants as the sole copy of Sup35 were transformed either with an empty vector (left) or with a plasmid expressing the matching Sup35 mutant under control of the *GAL1* promoter (right). All strains were cultured for three days in galactose/raffinose dropout medium, and then 10-fold serial dilutions were plated onto medium lacking adenine to select for [*PSI* ^+^].

The Sup35 nucleation domain shows a striking under-representation of highly hydrophobic residues, completely lacking F, I, L, M, W or V; however, it does contain five tyrosines. Three mutants were generated in which either two (−2TyrA and −2TyrB) or five of these tyrosines (−5Tyr) were eliminated from the nucleation domain (Figure 4A). Each was expressed at levels comparable to wild-type Sup35 (Figure 4B). None of these mutants showed detectable Ade^+^ colony formation when expressed at endogenous levels (Figure 4C).

However, because even wild-type Sup35 only rarely forms prions without Sup35 overexpression, it remained possible that the tyrosine deletion mutants were simply forming prions at a frequency below the threshold of detection. Indeed, transient over-expression of the corresponding PFD increased Ade^+^ colony formation by each of the strains (Figure 4C), suggesting that each of the mutants is capable of prion formation. However, the −5Tyr construct showed substantially reduced frequencies of Ade+ colony formation (Figure 4C). When tested for stability and curability, all Ade^+^ colonies isolated from the tyrosine deletion mutants were red after growth both with and without guanidine, indicating that the Ade^+^ phenotype is unstable (Figure S3).

**Figure 5:**
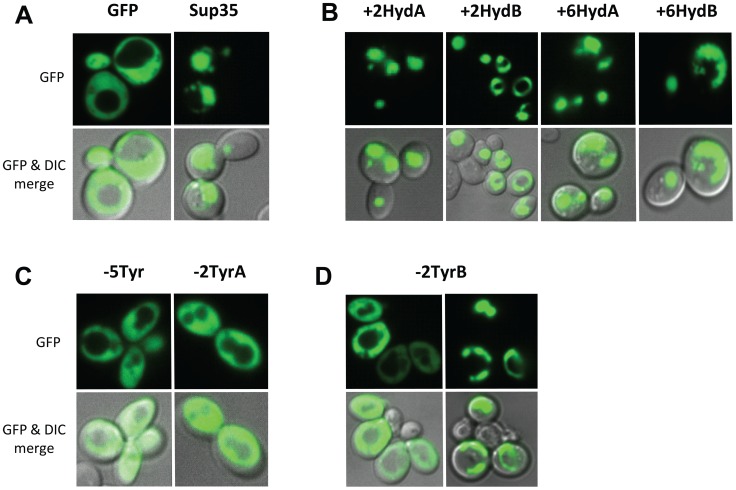
Tyrosine and hydrophobic residues promote foci formation. (A) The Sup35 PFD promotes formation of fluorescent foci. GFP or the NM domain from wild-type Sup35 fused to GFP were expressed under control of the *GAL1* promoter. Cells were grown in galactose/raffinose dropout medium for 24 h, and then visualized by confocal microscopy. (B) The hydrophobic insertion constructs each support formation of fluorescent foci. Conditions were as described in (A). (C) The −5Try and −2TyrA constructs fail to form fluorescent foci. (D) The −2TyrB construct forms foci in a fraction of cells.

Additionally, PFD-GFP fusions showed substantially reduced foci formation. For wild-type Sup35, over-expression of PFD-GFP fusions results in the formation of fluorescent foci (Figure 5A). Likewise, large foci were consistently observed for each of the hydrophobic addition constructs (Figure 5B). However, no foci were observed in cells expressing −2TyrA or −5Tyr (Figure 5C), while foci were observed in only a subset of the cells expressing −2TyrB (Figure 5D). Therefore, the tyrosine deletion mutants show substantially reduced *in vivo* aggregation, and appear completely unable to form stable [*PSI* ^+^] prions.

Some prion variants formed by wild-type Sup35 are either deleterious or lethal to yeast cells [65]. Therefore, the reduced prion formation and aggregation by the −5Tyr mutant could theoretically result from an artifact such as an increase in prion toxicity. However, the −5Tyr construct also showed substantially reduced aggregation kinetics *in vitro* (Figure 2), suggesting that tyrosine deletion directly affects aggregation propensity.

#### The effect of aromatic residues on prion formation

**Figure 6:**
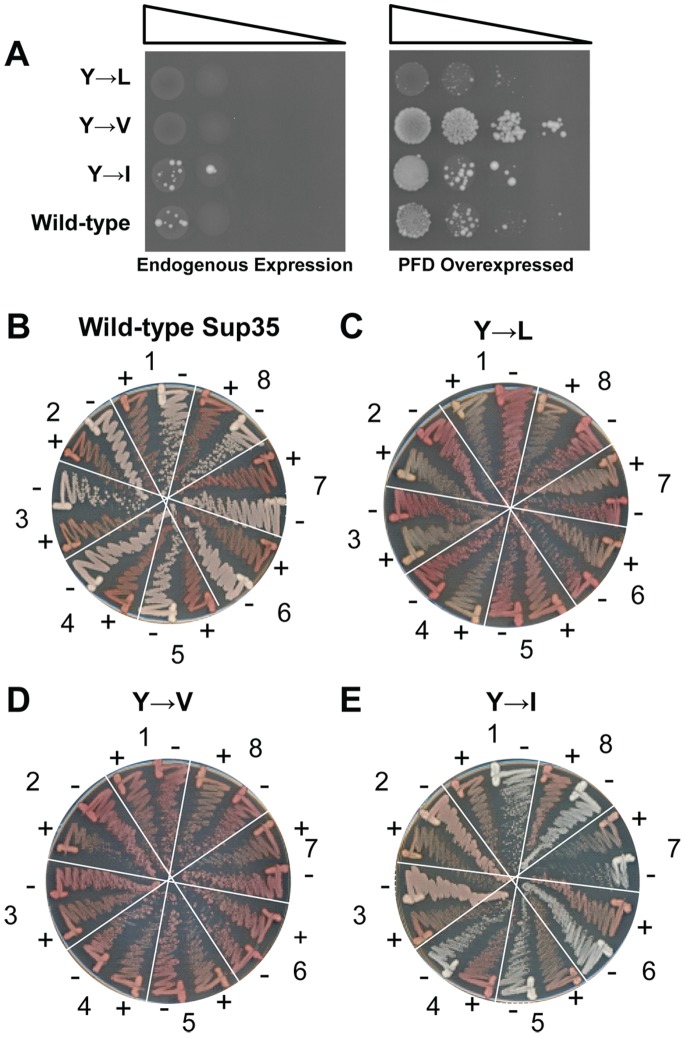
Aromatic residues are not required in the Sup35 nucleation domain. (A) The five tyrosines in the Sup35 nucleation domain (amino acids 1–40) were replaced with either leucines, isoleucines or valines. Strains were transformed either with an empty vector (left) or with a plasmid expressing the matching Sup35 mutant under control of the *GAL1* promoter (right). All strains were cultured for three days in galactose/raffinose dropout medium, and then 10-fold serial dilutions were plated onto medium lacking adenine to select for [*PSI* ^+^]. (B–E) Stability and curability of the Ade^+^ phenotype in cells expressing wild-type Sup35 (B), or Sup35 in which the five tyrosines in the nucleation domain were replaced with leucine (C), valine (D) or isoleucine (E). For each mutant, eight individual Ade^+^ isolates were grown on YPD (−) and YPD plus 4 mM guanidine HCl (+). Cells were then restreaked onto YPD to test for loss of the Ade^+^ phenotype.

Although there are very few highly hydrophobic residues in yeast PFDs, tyrosines are over-represented among a subset of yeast PFDs [16]. We directly compared the ability of hydrophobic and aromatic residues to drive prion formation by replacing the five native tyrosines in the Sup35 nucleation domain with leucines, isoleucines, or valines. All three constructs were able to form Ade^+^ colonies, albeit at different frequencies, and all three showed more Ade^+^ colony formation with PFD over-expression than without, consistent with the Ade^+^ colonies resulting from prion formation (Figure 6A). However, there were substantial differences both in the frequency of Ade^+^ colony formation (Figure 6A) and the fraction of these colonies that propagated as stable, curable prions (Figure 6B–E). The valine substitution construct showed the highest frequency of Ade^+^ colony formation (Figure 6A); however, the Ade^+^ colonies were consistently unstable, rapidly losing the Ade^+^ phenotype upon growth on non-selective medium (Figure 6D). While the construct with isoleucine substitutions showed less Ade^+^ colony formation, the majority of the Ade^+^ colonies were stable and curable (Figure 6E). The construct with leucine substitutions only formed small Ade^+^ colonies, and none of the Ade^+^ isolates were able to maintain the Ade^+^ phenotype upon non-selective growth (Figure 6C). This result is consistent with the relatively low prion propensity of leucine compared to the other hydrophobic residues [24], although it is possible that this failure to form stable prions is a result of a less direct effect, such as increased toxicity of prions formed by this mutant. Therefore, while the identity of the hydrophobic residues within the nucleation domain affects both prion formation and prion stability, there is no strict requirement for aromatic residues within the prion-nucleating domain of Sup35.

#### Compositional biases in glutamine/asparagine rich domains

The substantial effects of insertion or deletion of hydrophobic and aromatic residues in Sup35 highlight the narrow prion-propensity window required for a protein to act as a prion. According to PAPA, there are six strongly prion-promoting amino acids: F, I, V, Y, M and W. These amino acids have similar prion propensity scores, and are all predicted to be substantially more prion-prone than any other amino acid [24]. The 114-amino-acid Sup35 PFD contains 23 of these prion-promoting amino acids, representing 20.2% of the PFD; increasing this number to 24.2% in the +6Hyd constructs almost completely eliminated the soluble, functional state. It is likely that the exact number of prion-promoting residues required for prion activity is somewhat context-dependent; however, based on this dramatic effect of hydrophobic insertions, we hypothesized that it would be unlikely that any Q/N-rich regions in yeast would contain substantially more prion-promoting residues than Sup35.

**Figure 7:**
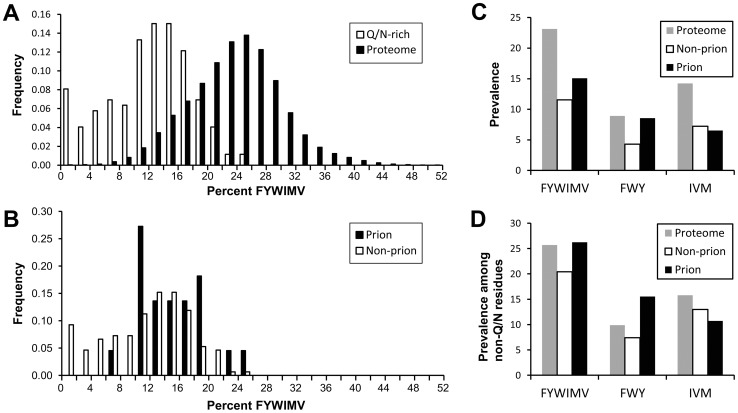
Amino acid composition of prion and non-prion Q/N-rich domains. (A) Histogram of the prevalence of strongly prion-promoting residues (FYWIMV) among Q/N-rich proteins (open bars) and among peptide fragments from the yeast proteome (black bars). For the Q/N-rich proteins, each of the regions of the yeast proteome identified by Harrison and Gerstein [16] as having high Q/N-bias were scored for the fraction of strongly prion-promoting amino acids. For the proteomic data, the yeast proteome was scanned using a 100 amino acid window size; each 100-amino-acid window was scored for the fraction of strongly prion-promoting amino acids. (B) Histogram of the prevalence of strongly prion-promoting amino acids among yeast prion and non-prion Q/N-rich domains. The black bars include Q/N-rich regions (as identified by Harrison and Gerstein) from yeast proteins shown to act as prions, as well as from proteins containing domains shown by Alberti *et al.* to have prion-like activity in four independent assays [19]. Open bars represent all other yeast Q/N-rich regions identified by Harrison and Gerstein. (C) Amino acid prevalence in Q/N-rich domains. Grey bars represent the prevalence of different groups of amino acids in the yeast proteome. Black bars represent the average frequency of these amino acids among Q/N-rich regions from both proteins shown to act as prions and proteins containing domains shown by Alberti *et al.* to have prion-like activity in four independent assays. Open bars represent the average frequency of these amino acids among all other yeast Q/N-rich domains identified by Harrison and Gerstein. (D) The prevalence of different groups of amino acids, plotted as a fraction of non-Q/N residues.

Indeed, this appears to be true. Harrison and Gerstein developed an algorithm to identify regions of high compositional bias [16]. They identified 170 regions in the yeast proteome with strong Q/N bias. There is substantial diversity in these regions; they vary from 25 to 886 amino acids long, and range from 16.8 to 96% Q/N content. Nevertheless, only one of these 170 Q/N-rich domains has more than the 24.2% F, I, V, Y, M and W that is found in the +6Hyd constructs (Figure 7A); the lone exception is a fragment from New1, which has 25.5%. By contrast, when the yeast proteome is scanned with a 100-amino-acid window size, over half of all protein fragments have more than the 24.2% F, I, V, Y, M and W. Moreover, although F, I, V, Y, M and W constitute 23.1% of the yeast proteome, the Q/N-rich regions contain on average only 11.9% of these residues.

There are also subtle differences in the frequencies of strongly prion-promoting residues between the prion-forming and non-prion Q/N-rich domains. The Harrison and Gerstein data set includes fragments from 22 proteins with clear prion activity. Eight of these have been proven to act as prions, while an additional fourteen were shown by Alberti *et al.* to have prion-like activity in four independent assays [19]. The non-prion Q/N-rich sequences show both lower average frequencies of strongly prion-promoting residues (Figure 7C) and a broader range (Figure 7B). Additionally, there appear to be differences in which strongly prion-promoting residues are found in the prion versus non-prion sequences. While the two sets have similar numbers of non-aromatic prion-promoting residues (I, V and M), the prion sequences have substantially more aromatic residues (Figure 7C).

The substantial bias against strongly prion-promoting residues in non-prion Q/N-rich domains could simply be a result of high Q/N content. Because these domains average about 45% Q/N residues, the high Q/N content may simply crowd out other residues. However, when the frequency of strongly prion promoting residues is calculated as a percentage of the total number of non-Q/N residues, the prion-promoting amino acids are still slightly under-represented in non-prion Q/N-rich domains. In the yeast genome, F, I, V, Y, M and W constitute 25.7% of the non-Q/N residues (Figure 7D). This is similar to their average frequency in Q/N-rich PFDs; by contrast, the average among non-prion Q/N-rich domains is 20.4%.

There are other datasets of Q/N-rich proteins that could have been used for the analysis in Figure 7, each with unique strengths and weaknesses. The Harrison and Gerstein data set is useful, because it identifies regions with strong Q/N-bias without imposing any additional compositional requirements. Nevertheless, it has the disadvantage that, because it simply identifies regions with strong statistical bias for Q/N residues, it includes some very large regions that are only modestly enriched for Q/N. However, very similar results were observed with a second data set; Michelitsch and Weissman developed a search algorithm called DIANA. DIANA identified every yeast protein that contains an 80 residue window with at least 30 Q/Ns; then, within these proteins it identified the most Q/N-rich 80 amino-acid segment. While this method is effective for identifying prion candidates, it was not as ideal for our purposes; because the window size is fixed at 80 amino acids, in some cases only a portion of the 80 amino acid segment is Q/N-rich, while in other cases the algorithm may capture only a portion of a long Q/N-rich segment. Nevertheless, the same basic trends were seen in this data set as in the Harrison and Gerstein set (Figure S4).

## Discussion

We previously scored the prion propensity of each amino acid in the context of a Q/N-rich PFD [24], and were surprised to find that there was little correlation between the amino acids that most strongly support prion formation and those that are actually found in yeast PFDs. Here, we provide an explanation for this apparent contradiction.

We first confirmed that non-aromatic hydrophobic residues do strongly promote prion formation. We found that both aromatic residues and non-aromatic hydrophobic residues (with the exception of leucine) all promote prion nucleation, albeit to varying degrees, demonstrating that our previous prion-propensity estimates were not an artifact of the region tested or a product of sampling error. This effect was even stronger than we anticipated, and suggests that prion formation can easily be controlled by modifying the number and position of hydrophobic residues. However, the question remained, if these residues promote prion formation, why are they so rare in actual PFDs?

Combined with our experimental data, our bioinformatics analysis suggests an answer to this question. Strongly prion-promoting residues (F, W, Y, I, V and M) are under-represented among both prion and non-prion Q/N-rich domains (Figure 7), most likely because too many of these residues would make proteins excessively aggregation-prone. A variety of evidence indicates that aromatic residues facilitate prion maintenance [33], [43]. This requirement for aromatic residues, coupled with a limit on the number of strongly prion-promoting residues tolerated in Q/N-rich domains, likely leads to the exclusion of non-aromatic residues from yeast PFDs. It should be noted that one study suggests that another difference between aromatic and non-aromatic hydrophobics is that aromatic residues, but not non-aromatic hydrophobic residues, can make contacts that facilitate the early oligomerization steps in prion formation [66]. However, in that study, leucine was used as the non-aromatic hydrophobic residue; leucine is uniquely non-prion-prone among the hydrophobic residues [24], presumably due to its low β-sheet propensity [67], so additional studies will be needed to determine whether this result applies to all hydrophobic residues. Regardless, our results strongly argue that hydrophobic residues are rare not because they inhibit prion formation, but because aromatic residues are equally able to support prion formation, and can also contribute to other steps in prion activity.

Indeed, while it has been well-documented that non-aromatic hydrophobic residues are under-represented in yeast PFDs [16], [68], the fact that these residues are almost equally rare among non-prion Q/N-rich domains is often ignored. This highlights a key point: the sequence features that most clearly distinguish Q/N-rich PFDs from the entire proteome may not be the same features that most effectively distinguish between prion and non-prion Q/N-rich domains. This distinction likely explains why algorithms designed to identify new prions based on compositional similarity to existing prions are very effective at identifying prion candidates, yet are far less effective at ranking the top candidates [24]. Unfortunately, this distinction continues to be missed. For example, a recent paper by Espinosa Angarica *et al.* argued that because C, W and E are rare among yeast PFDs, these residues must make an “unfavorable contribution” to prion activity [68]; however, this analysis ignores the fact that while W is rare among yeast PFDs, it is even more rare among non-prion Q/N-rich domains.

One key caveat with these experiments is that different regions of PFDs may have different sequence requirements, based on their respective roles in prion activity. For Sup35, the nucleation domain and remainder of the prion domain (termed the oligopeptide repeat domain, due to the presence of a series of imperfect peptide repeats) have both distinct roles in prion activity [44] and distinct compositional requirements [34]. By focusing on the nucleation domain, we were able to specifically isolate the effects of hydrophobic residues on prion formation. In the nucleation domain, non-aromatic hydrophobic residues and aromatic residues seem at least partially interchangeable. However, we have separately begun to systematically examine the distinct sequence requirements for prion formation versus maintenance, and have found that aromatic residues appear to play a more essential role within the ORD (unpublished data), consistent with their proposed functions in prion maintenance [33], [43].

Our results explain a number of other conundrums in the prion field. There has been substantial debate about the role of short sequence motifs in yeast prion formation. For example, a variety of evidence suggests that a short segment of the Sup35 PFD spanning amino acids 8–24 acts as a key nucleating site for prion formation, and point mutations in this region can prevent addition to prion aggregates and can substantially affect efficiency of cross-species transmission [58], [60]. However, much larger fragments are required for prion activity [44]; the shortest region from any prion protein that has be shown to support prion activity is 37 amino acids from Swi1 [69]. Furthermore, the fact that PFDs can be scrambled without blocking prion formation seems to argue against the importance of short sequence motifs. Our data provide a simple explanation for this apparent contradiction. If yeast PFDs contain relatively few strongly prion-promoting amino acids, then wherever these amino acids are located will naturally act as potential nucleating sites. Indeed, residues 8–24 of Sup35 contain two strongly prion promoting amino acids, and no strongly inhibiting amino acids (charged residues or prolines). In fact, the longest segment in the Sup35 PFD without a prion-inhibiting amino acid spans residues 4–27. Thus, the key role of this segment in prion nucleation may be explainable solely based on composition.

Interestingly, while composition is the dominant factor in determining prion activity, our data clearly demonstrate that primary sequence can exert a substantial effect on both the frequency of prion formation and the stability of the prion phenotype. The basis for this effect is unclear. Composition-based algorithms such as PAPA clearly do not predict such a strong effect of primary sequence. However, even algorithms designed to detect primary-sequence motifs appear to be no more effective (Figure 3B). While this set of mutants is likely too small to extract the exact relationship between primary sequence and prion propensity, this data set could provide a useful tool for testing future primary-sequence-based algorithms. However, it is important to note that the observed differences may not be due solely to differences in prion propensity. Because some prion variants can be deleterious or lethal [65], a mutation that shifts the distribution of variants formed by the protein to more toxic variants (or increases the toxicity of common variants) could give the appearance of reducing prion propensity. Therefore, more detailed studies will be needed to untangle the basis for this primary sequence effect.

## Supporting Information

Figure S1 **Strain YER632/pJ533 was streaked for three consecutive passages on YPAD +4 mM guanidine HCl.** This strain was then transformed with plasmids expressing the indicated Sup35 mutants. After FOA selection for loss of pJ533, the strains were transformed with empty vector (left) or with a plasmid expressing the matching Sup35 mutant under control of the *GAL1* promoter (right). All strains were cultured for three days in galactose/raffinose dropout medium, and then 10-fold serial dilutions were plated onto medium lacking adenine to select for [*PSI* ^+^]. As a control, prion formation by YER632/pJ533 before (wild-type, [*PIN* ^+^]) and after (wild-type, [*pin* ^−^]) guanidine treatment is shown.
(TIF)

Figure S2 **Hydrophobic addition constructs form curable prions.** For +2HydA (A), +2HydB (B), +6HydA (C), and +6HydB (D), eight individual Ade^+^ isolates were grown on YPD (−) and YPD plus 4 mM guanidine HCl (+). Cells were then restreaked onto YPD to test for loss of the Ade^+^ phenotype.
(TIF)

Figure S3 **Stability and curability of Ade^+^ colonies formed by tyrosine deletion constructs.** For −5Tyr (A), −2TyrA (B), and −2TyrB (C), eight individual Ade^+^ isolates were grown on YPD (−) and YPD plus 4 mM guanidine HCl (+). Cells were then restreaked onto YPD to test for loss of the Ade^+^ phenotype.
(TIF)

Figure S4 **Amino acid composition of prion and non-prion Q/N-rich domains, using the data set of Michelitsch and Weissman.** (A) Histogram of the prevalence of strongly prion-promoting amino acids (FYWIMV) among regions of the yeast proteome identified by Michelitsch and Weissman as being highly enriched in Q/N-residues. The black bars include Q/N-rich regions from proteins shown to act as prions, as well as from proteins containing domains shown by Alberti *et al.* to have prion-like activity in four independent assays. Open bars represent all other Q/N-rich regions identified by Michelitsch and Weissman. (B) The prevalence of different groups of amino acids in the yeast genome (grey bars) compared to the average frequency of these amino acids among Q/N-rich prion (black bars) and non-prion (open bars). (C) The prevalence of different groups of amino acids, plotted as a fraction of non-Q/N residues.
(TIF)

Table S1 **Oligonucleotides used in this study.**
(DOCX)
