# Supplementary figures and images for: Increasing Prion Propensity by Hydrophobic Insertion
Source: PLoS One. 2014 Feb 20;9(2):e89286. doi: 10.1371/journal.pone.0089286 (PMC3930707; doi:10.1371/journal.pone.0089286)

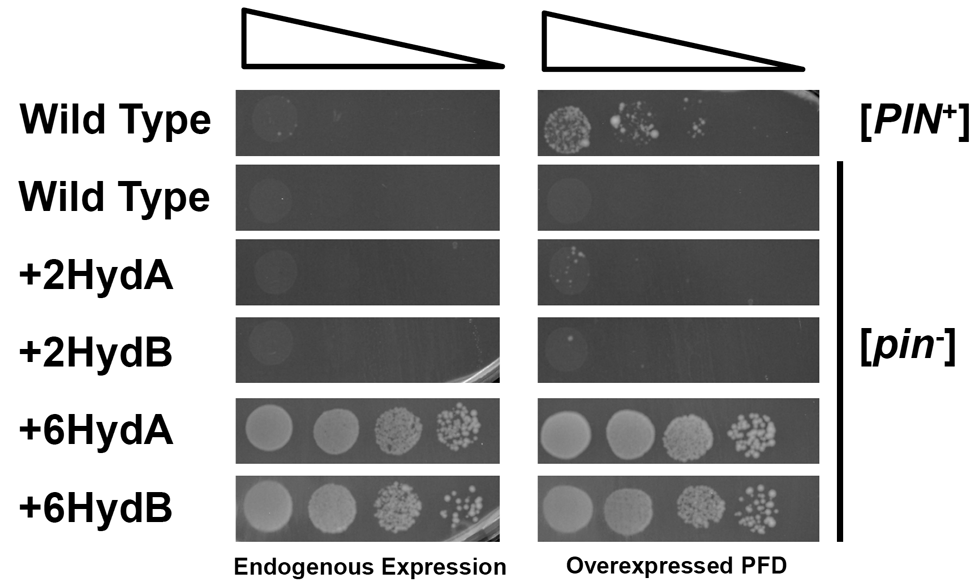

Supplement: Figure S1 — Strain YER632/pJ533 was streaked for three consecutive passages on YPAD +4 mM guanidine HCl. This strain was then transformed with plasmids expressing the indicated Sup35 mutants. After FOA selection for loss of pJ533, the strains were transformed with empty vector (left) or with a plasmid expressing the matching Sup35 mutant under control of the GAL1 promoter (right). All strains were cultured for three days in galactose/raffinose dropout medium, and then 10-fold serial dilutions were plated onto medium lacking adenine to select for [PSI +]. As a control, prion formation by YER632/pJ533 before (wild-type, [PIN +]) and after (wild-type, [pin −]) guanidine treatment is shown. (TIF) [file pone.0089286.s001.tif]

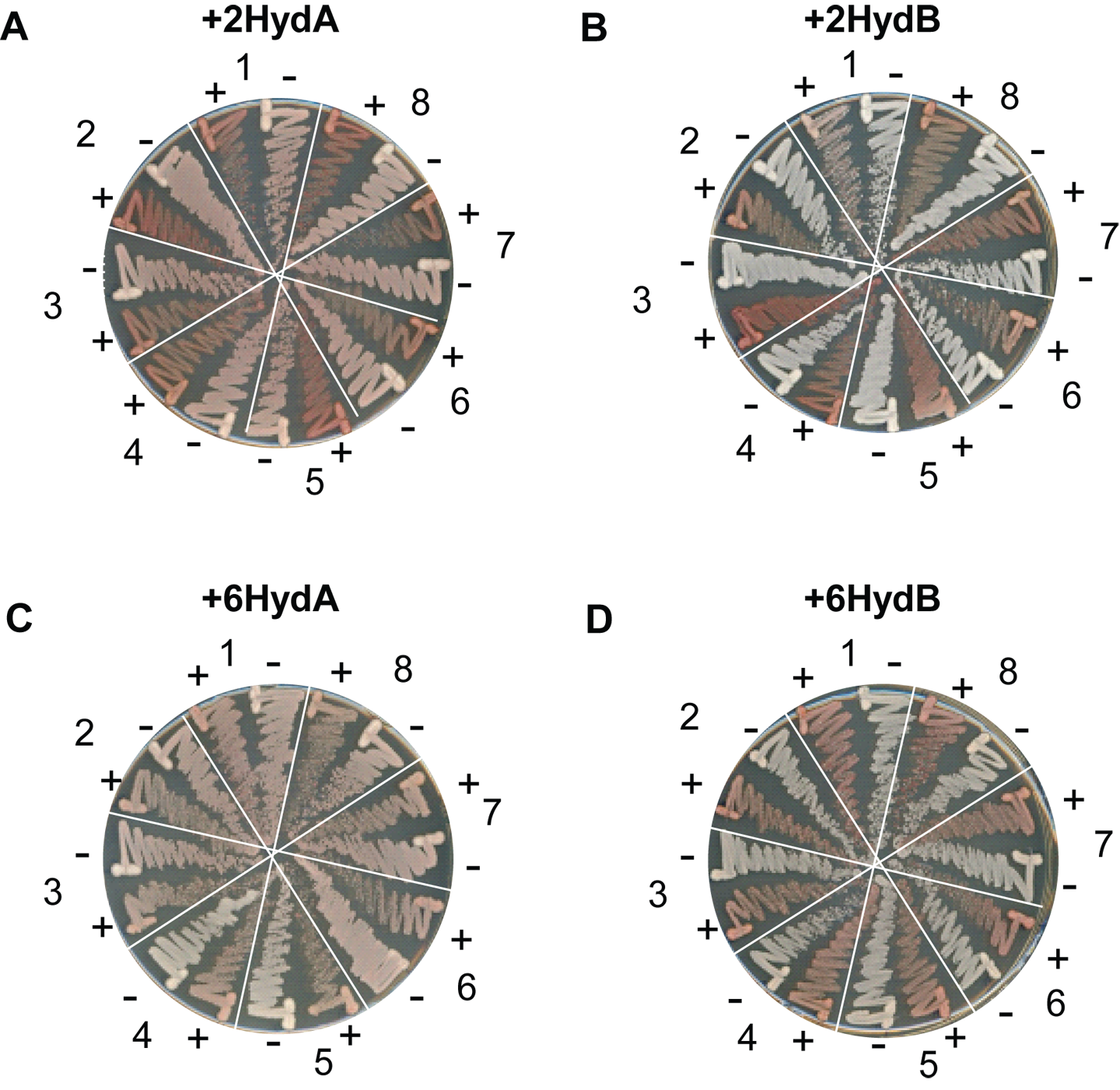

Supplement: Figure S2 — Hydrophobic addition constructs form curable prions. For +2HydA (A), +2HydB (B), +6HydA (C), and +6HydB (D), eight individual Ade+ isolates were grown on YPD (−) and YPD plus 4 mM guanidine HCl (+). Cells were then restreaked onto YPD to test for loss of the Ade+ phenotype. (TIF) [file pone.0089286.s002.tif]

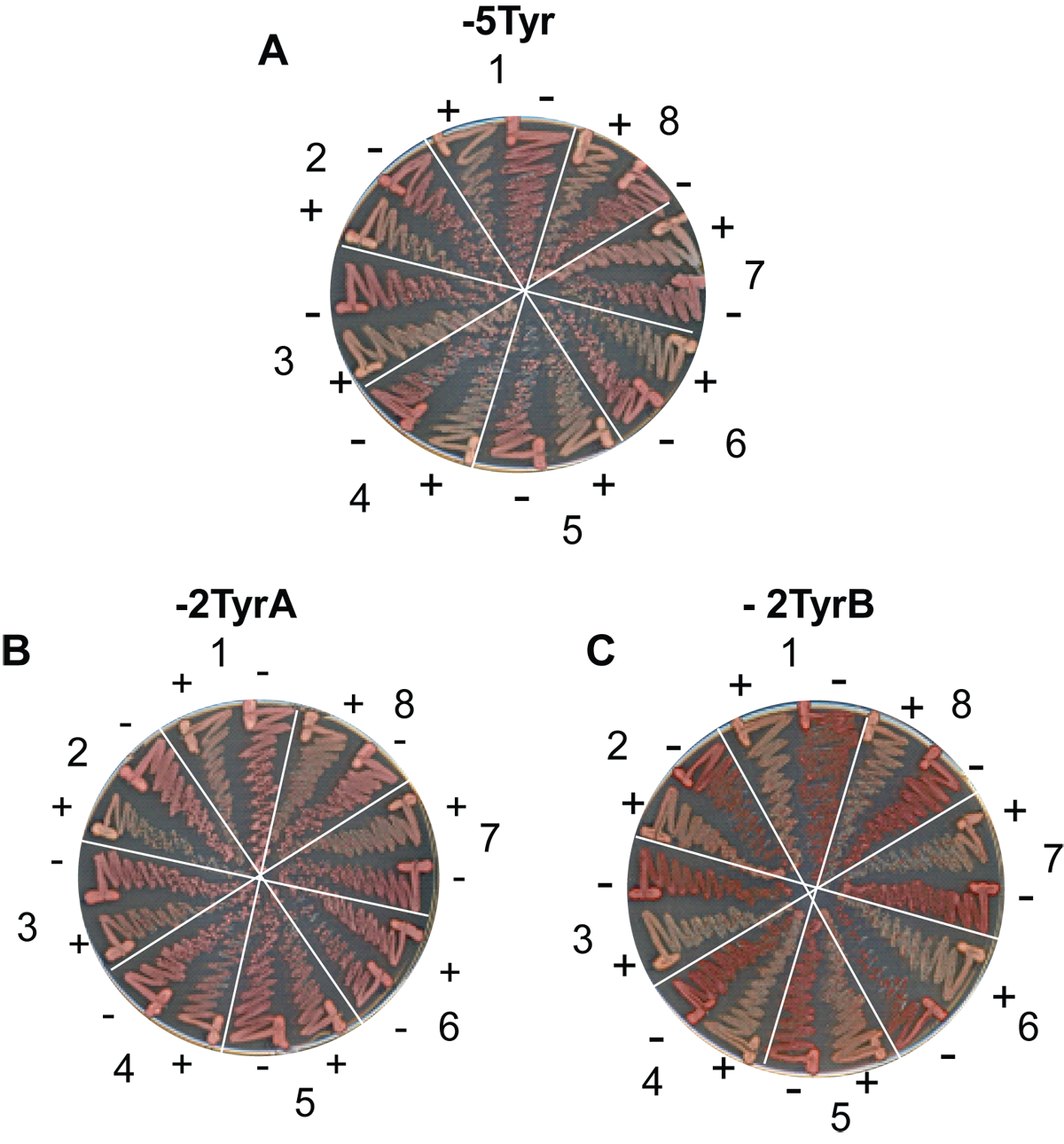

Supplement: Figure S3 — Stability and curability of Ade+ colonies formed by tyrosine deletion constructs. For −5Tyr (A), −2TyrA (B), and −2TyrB (C), eight individual Ade+ isolates were grown on YPD (−) and YPD plus 4 mM guanidine HCl (+). Cells were then restreaked onto YPD to test for loss of the Ade+ phenotype. (TIF) [file pone.0089286.s003.tif]

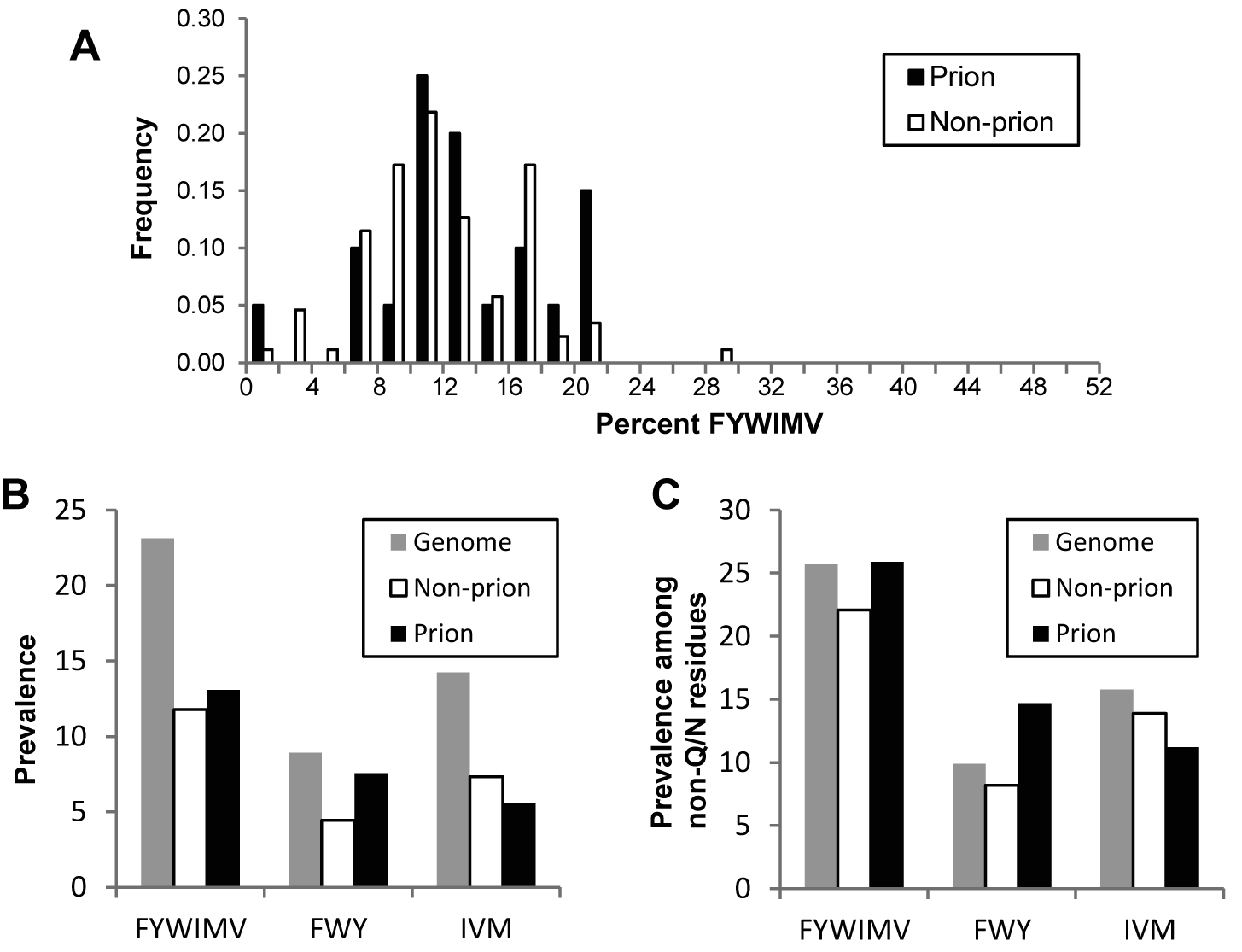

Supplement: Figure S4 — Amino acid composition of prion and non-prion Q/N-rich domains, using the data set of Michelitsch and Weissman. (A) Histogram of the prevalence of strongly prion-promoting amino acids (FYWIMV) among regions of the yeast proteome identified by Michelitsch and Weissman as being highly enriched in Q/N-residues. The black bars include Q/N-rich regions from proteins shown to act as prions, as well as from proteins containing domains shown by Alberti et al. to have prion-like activity in four independent assays. Open bars represent all other Q/N-rich regions identified by Michelitsch and Weissman. (B) The prevalence of different groups of amino acids in the yeast genome (grey bars) compared to the average frequency of these amino acids among Q/N-rich prion (black bars) and non-prion (open bars). (C) The prevalence of different groups of amino acids, plotted as a fraction of non-Q/N residues. (TIF) [file pone.0089286.s004.tif]
