# Supplementary material for: Increasing Prion Propensity by Hydrophobic Insertion
Source: PLoS One. 2014 Feb 20;9(2):e89286. doi: 10.1371/journal.pone.0089286 (PMC3930707; doi:10.1371/journal.pone.0089286)
Supplement: Table S1 — Oligonucleotides used in this study. (DOCX) [file pone.0089286.s005.docx]

**Table S1: Oligonucleotides used in this study**

| **Name** | **Description** | **Sequence** |
| --- | --- | --- |
|  |  |  |
| EDR1263 | Sense primer to build +2HydA | GTCGGATTCAAACCAAGGCAACAATATCCAGCAAAACTACCAGCAATACAGCCAGAACGGTAACGTTCAACAACAAGGTAACAACAGATACC |
| EDR1264 | Antisense primer to build +2HydA, C-I | GTTGCCTTGGTTTGAATCCGAC |
| EDR1265 | Sense primer to build +2HydB | GGATTCAAACCAAGGCAACAATCAGCAAGTCAACTACCAGCAATACAGCCAGATTAACGGTAACCAACAACAAGGTAACAACAGATACC |
| EDR1266 | Antisense primer to build +2HydB | GACTTGCTGATTGTTGCCTTGGTTTGAATCC |
| EDR1267 | Sense primer to build +6HydA | GTCGGATTCAAACCAAGGCAACATCAATCAGCAAATTAACTACCAGGTTCAATACATTAGCGTCCAGGTTAACGGTAACCAACAACAAGGTAACAACAG |
| EDR1268 | Antisense primer to build +6HydA | GTTGCCTTGGTTTGAATCCG |
| EDR1269 | Sense primer to build +6HydB | GGCAACATCAATCAGCAAGTCAACGTTTACCAGCAATACAGCCAGAACGGTAACGTTCAAATTATCCAACAAGGTAACAACAGATACCAAGG |
| EDR1270 | Antisense primer to build +6HydB | GACTTGCTGATTGATGTTGCCTTGGTTTGAATCCGAC |
| EDR1257 | Sense primer to build -5Tyr | GCAACAATCAGCAAAACCAGCAAAGCCAGAACGGTAACCAACAACAAGGTAACAACAGACAAGGTCAAGCTAATGCTCAAGCCCAACCTGCAG |
| EDR1258 | Antisense primer to build -5Tyr | GCTTTGCTGGTTTTGCTGATTGTTGCCTTGGTTTGAATCC |
| EDR1259 | Sense primer to build -2TyrA | CAAACCAAGGCAACAATCAGCAAAACCAGCAATACAGCCAGAACGGTAACCAACAACAAGGTAACAACAGACAAGGTTATCAAGCTTACAATGCTCAAGC |
| EDR1260 | Antisense primer to build -2TyrA | GTTTTGCTGATTGTTGCCTTGGTTTGAATCC |
| EDR1261 | Sense primer to build -2TyrB | GCAACAATCAGCAAAACTACCAGCAAAGCCAGAACGGTAACCAACAACAAGGTAACAACAGATACCAAGGTCAAGCTTACAATGCTCAAGCCC |
| EDR1262 | Antisense primer to build -2TyrB | GGCTTTGCTGGTAGTTTTGCTGATTGTTGC |
| EDR1409 | Sense primer to build +2HydC | GTCGGATTCAAACCAAGGCAACAATGTTCAGCAAAACTACCAGCAATACAGCCAGAACGGTAACATCCAACAACAAGGTAACAACAGATACC |
| EDR1403 | Sense primer to build +2HydD | GTCGGATTCAAACCAAGGCAACAATCAGCAAATCAACTACCAGCAATACAGCCAGGTTAACGGTAACCAACAACAAGGTAACAACAGATACC |
| EDR1404 | Sense primer to build +2HydE | GTCGGATTCAAACCAAGGCAACAATCAGCAAATCAACTACCAGCAATACAGCCAGAACGTTGGTAACCAACAACAAGGTAACAACAGATACC |
| EDR1405 | Sense primer to build +2HydF | GTCGGATTCAAACCAAGGCAACAATCAGCAAATCAACTACCAGCAATACAGCCAGAACGGTGTTAACCAACAACAAGGTAACAACAGATACC |
| EDR1406 | Sense primer to build +2HydG | GTCGGATTCAAACCAAGGCAACAATCAGCAAATCAACTACCAGCAATACAGCCAGAACGGTAACGTTCAACAACAAGGTAACAACAGATACC |
| EDR1407 | Sense primer to build +2HydH | GTCGGATTCAAACCAAGGCAACAATCAGATCCAAAACTACCAGCAATACAGCCAGGTTAACGGTAACCAACAACAAGGTAACAACAGATACC |
| EDR1408 | Sense primer to build +2HydI | GTCGGATTCAAACCAAGGCAACAATATCCAGCAAAACTACCAGCAATACAGCCAGGTTAACGGTAACCAACAACAAGGTAACAACAGATACC |
| EDR1308 | Sense primer to build Sup35(Y→L) | CAGATTGCAAGGTTTACAAGCTCTGAATGCTCAAGCCCAACCTGCAG |
| EDR1309 | Antisense primer to build Sup35(Y→L) | CAGAGCTTGTAAACCTTGCAATCTGTTGTTACCTTGTTGTTGGTTACCGTTCTGGCTCAATTGCTGTAAGTTTTGCTGATTGTTGCCTTGGTTTGAATCC |
| EDR1310 | Sense primer to build Sup35(Y→V) | CAGAGTTCAAGGTGTACAAGCTGTGAATGCTCAAGCCCAACCTGCAG |
| EDR1311 | Antisense primer to build Sup35(Y→V) | CACAGCTTGTACACCTTGAACTCTGTTGTTACCTTGTTGTTGGTTACCGTTCTGGCTAACTTGCTGAACGTTTTGCTGATTGTTGCCTTGGTTTGAATCC |
| EDR1312 | Sense primer to build Sup35(Y→I) | CAGAATCCAGGGTATTCAAGCTATCAATGCTCAAGCCCAACCTGCAG |
| EDR1313 | Antisense primer to build Sup35(Y→I) | GATAGCTTGAATACCCTGGATTCTGTTGTTACCTTGTTGTTGGTTACCGTTCTGGCTTATTTGCTGAATGTTTTGCTGATTGTTGCCTTGGTTTGAATCC |
| EDR1747 | Sense primer to make pER687 | GCTTCCAATGATGCATGCATGGACGCAAAGAAGTTTAATAATCATATTACATGG |
| EDR1748 | Antisense primer to make pER687 | GGACCTCAAGATGAATTCCCATTCAGGCTGCGCAACTG |
| EDR1898 | Sense primer to make pER760 | GAGCTACTGGATCCACAATGTCTGGAGCTCCCTCGAGTGGAGGTAGCTACTCTAAAGGTGAAGAATTATTCACTGGTG |
| EDR1899 | Antisense primer to make pER760 | GGATGTCAGTTGTCGACTTTATTTGTACAATTCATCCATACCATGGG |
| EDR1924 | Antisense primer to make NM-GFP fusions | GTCGATGCTACTCGAGTCGTTAACAACTTCGTCATCCACTTC |
| EDR1084 | Common primer to build induction plasmids and NM-GFP fusions | CGATGCTACTCGAGTTTACATATCGTTAACAACTTCGTCATCCAC |
| EDR1008 | Common primer to build induction plasmids | GAGCTACTGGATCCACAATGTCGGATTCAAACCAAGGCAAC |
| EDR262 | Antisense primer to Sup35 | GCATCAGCACTGGTAACATTGG |
| EDR301 | Sense primer binding upstream of Sup35 | CGTCACAGTGTTCGAGTCTG |
|  |  |  |
|  |  |  |
|  |  |  |
